# Hand preference for the visual and auditory modalities in humans

**DOI:** 10.1038/s41598-021-87396-4

**Published:** 2021-04-12

**Authors:** Yuqian Yang, Peter H. Weiss, Gereon R. Fink, Qi Chen

**Affiliations:** 1 Cognitive Neuroscience, Institute of Neuroscience and Medicine (INM-3), Research Centre Jülich, Wilhelm-Johnen-Str., 52428 Jülich, Germany; 2 Department of Neurology, University Hospital Cologne and Faculty of Medicine, University of Cologne, 50937 Cologne, Germany; 3 Center for Studies of Psychological Application and School of Psychology, South China Normal University, Guangzhou, 510631 China; 4 Key Laboratory of Brain, Cognition and Education Sciences (South China Normal University), Ministry of Education, Guangzhou, 510631 China

**Keywords:** Neuroscience, Psychology

## Abstract

The sensory dominance effect refers to the phenomenon that one sensory modality more frequently receives preferential processing (and eventually dominates consciousness and behavior) over and above other modalities. On the other hand, hand dominance is an innate aspect of the human motor system. To investigate how the sensory dominance effect interacts with hand dominance, we applied the adapted Colavita paradigm and recruited a large cohort of healthy right-handed participants (n = 119). While the visual dominance effect in bimodal trials was observed for the whole group (n = 119), about half of the right-handers (48%) showed a visual preference, i.e., their dominant hand effect manifested in responding to the visual stimuli. By contrast, 39% of the right-handers exhibited an auditory preference, i.e., the dominant hand effect occurred for the auditory responses. The remaining participants (13%) did not show any dominant hand preference for either visual or auditory responses. For the first time, the current behavioral data revealed that human beings possess a characteristic and persistent preferential link between different sensory modalities and the dominant vs. non-dominant hand. Whenever this preferential link between the sensory and the motor system was adopted, one dominance effect peaks upon the other dominance effect’s best performance.

## Introduction

The human brain continually receives streams of information from multiple sensory modalities, either complementary or conflicting. In such a context, selective attention needs to crossmodally coordinate sensory percepts. Accordingly, robust cross-modal links in spatial attention have been demonstrated between visual, auditory, and tactile stimuli^1–3^. For example, an irrelevant touch, which simultaneously appears and shares the spatial location with a visual target, can improve vision and enhance neural activity in the early visual cortex^3^. On the other hand, in the non-spatial domain, cross-modal competition happens when our brain cannot assign equal attentional resources to diverse sensory inputs, depending on the current behavioral goals^4,5^. Therefore, information from one sensory modality can dominate or even extinct the explicit sensory processing of other modalities, referred to as ‘sensory dominance’^4–8^. One striking example of the sensory dominance effect is the Colavita visual dominance effect. Participants often fail to respond to the auditory component of simultaneously presented bimodal audiovisual stimuli and are even unaware of such bimodal trials^9^. The classical theoretical accounts on the crossmodal link in spatial attention^1–3^ and on the sensory dominance effect^4,5,10^ primarily focus on the stimulus/sensory property domain and do not refer to the response codes. However, please note that to perform a behavioral task successfully, all bottom-up sensory inputs from different modalities need to be transformed into their corresponding sensorimotor representations, e.g., via the visual or the auditory dorsal pathway^11–15^. It remains poorly understood how the sensorimotor processes contribute to the sensory dominance effect.

Just as the sensory dominance effect between the different sensory systems, the hand dominance effect is one essential characteristic of hemispheric specialization in the motor system^16^. Independent of cultural and historical backgrounds, around 90% of humans prefer to use their right hand for skilled manipulation, i.e., show right-handedness^17^. Consequently, task performance with the dominant hand is often superior to that with the non-dominant hand^18–24^. By associating different sensory inputs (visual vs. auditory modality) with different underlying sensorimotor codes (dominant vs. non-dominant hand response), we aimed to investigate how the sensory dominance effect interacts with the hand dominance effect. Specifically, we orthogonally combined the hand dominance effect with the sensory dominance effect in an adapted version of the Colavita paradigm^25–28^. The adapted Colavita paradigm measures the size of the visual dominance and the auditory dominance effects in terms of both the proportions of the visual vs. auditory dominance trials and the reaction times (RTs) in the bimodal trials^25–28^. The correspondence between the sensory modality (visual vs. auditory) and the response hand (dominant vs. non-dominant) was fully counterbalanced within each participant. Moreover, a large sample (n = 119) of healthy right-handed subjects were recruited to ensure the generalization of the present results across the population.

Notably, no consistent and stable RT advantage of the dominant (right) over the non-dominant (left) hand has been previously reported for right-handers when responding to visual and auditory stimuli. Although some previous studies reported a dominant hand benefit to both visual and auditory stimuli^29,30^, no significant dominant hand advantage was reported for either auditory or visual stimuli in other studies^23,31–33^. These ambiguous data led to the notion that there might be substantial individual differences in the human population concerning the dominant hand’s processing efficiency for the auditory versus visual stimuli^34–37^. However, no systematic studies have been performed to investigate individual differences in the preferential link between the dominant vs. non-dominant hand and the visual vs. auditory modality. The present study’s hypothesis was that each human being has her/his characteristic and stable hand-modality preference, and this hand-modality preference varies between individuals. Some people might exhibit a dominant hand advantage for the visual modality, while other people show a dominant hand advantage for the auditory modality. Therefore, no consistent dominant-hand benefits will be found at the group level, for neither the visual nor the auditory modality, when data from right-handers with different hand-modality preferences are collapsed.

Furthermore, it remains to be elucidated how the hand-modality preference interacts with the sensory dominance effect. Especially when a sensory modality is paired with its preferred hand, the corresponding sensory information can access its sensorimotor representations faster. It will thus more efficiently dominate the other sensory modality than when paired with its non-preferred hand. Similarly, when the dominant-hand is paired with its preferred sensory modality, the efficiency of the dominant motor system (i.e., especially the motor cortex in the left hemisphere of the right-handers) is likely to be amplified so that the dominant hand benefit will increase.

## Materials and methods

### Participants

A total of 119 healthy right-handed participants (18–25 years old) volunteered for the study. They had normal hearing and vision and not ever experienced neurological or psychiatric illness before. Before the experiment, all participants had signed their informed consent based on the Helsinki Declaration and been rewarded as compensation. The study was conducted under the approval of the Ethics Committee of the School of Psychology, South China Normal University.

### Handedness assessment

Handedness was assessed using the Edinburgh-Handedness-Inventory with 10 items (EHI)^38^. In the EHI, participants rated their hand-preference strength for ten daily activities (e.g., writing, drawing, throwing). The lateralization quotient (LQ) ranging from − 100 to 100 indicates the direction and degree of the hand preference: an LQ more than 25 indicates right-handedness; an LQ less than − 25 indicates left-handedness^39^. The mean LQ of the right-handed participants in the present study was 77 (SD = 7).

### Stimuli and experimental design

#### Stimuli and procedure

**Figure 1:**
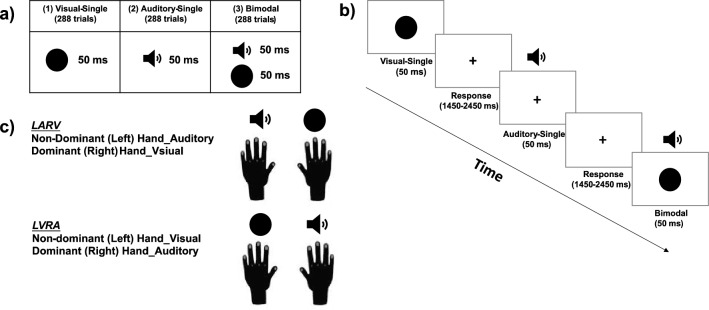
Experimental design (**a**) stimuli in the adapted Colavita paradigm adopted in the current study. Three types of stimuli were used: the unimodal visual target, the unimodal auditory target, and the bimodal audiovisual target. (**b**) Schematic depiction of the time course starting with a unimodal visual trial followed by a unimodal auditory trial and a bimodal trial. (**c**) The correspondence between the sensory modality and the response hand was counterbalanced within each subject. Participants used their non-dominant (left) hand to respond to the auditory target and their dominant (right) hand to respond to the visual target (LARV) for the first half of the trials and vice versa (LVRA) for the second half of the trials. The order of the two kinds of the modality-hand assignment was counterbalanced across subjects.

The current study applied the same adapted Colavita paradigm and target stimuli as those in the previous supportive literature^26,28^. Specifically, a pure tone with a frequency of 4000 Hz and a comfortable loudness level (approximately 68–72 dB) delivered via headphones (Sennheiser HD 202 II) for 50 ms served as the auditory target. And a black sphere (red–green–blue value, 0, 0, and 0) with a visual angle of 1.5° and a luminance of 1.9 cd/m^2^ shown at the center of an LCD monitor for 50 ms served as the visual target. Besides, a black cross of 1° (horizontally) × 1° (vertically) visual angle shown at the center of a white background was the default visual display (red–green–blue value, 255, 255, and 255; Fig. 1a). The visual and auditory targets were presented either independently (i.e., the Visual-Single and the Auditory-Single trials) or simultaneously (i.e., the bimodal trials). The three types of trials (288 of each type) were mixed randomly in the formal experiment. Both unimodal and bimodal trials lasted for 1500 ms (= 50 ms stimulus duration plus 1450 ms time window for response) and were jittered between 4 levels from 200 to 1000 ms (Fig. 1b). The experiment took approximately 40 min to complete in total.

Participants were tested in a dimly lighted and soundproof room and were required to fixate the central cross during the entire experiment. Their task was to press the pre-specified buttons with their index fingers as quickly and accurately as possible whenever the target stimuli were presented. Moreover, in order to derive the responses of both the dominant (right) the non-dominant (left) hand for both the visual and auditory responses in either the unimodal or the bimodal conditions, two kinds of hand-modality assignments^26^ were adopted within participants. For half of the trials (n = 432, including 144 of each type of trial), participants were instructed to respond to the auditory target with their non-dominant (left) hand and the visual target with their dominant (right) hand (i.e., Left-Auditory-Right-Visual [= LARV] assignment), and to reverse the mapping between the visual and auditory targets and the two hands for the other half of trials (i.e., Left-Visual-Right-Auditory [= LVRA] assignment)^26^. The two kinds of hand-modality assignments were blocked, and their order was counterbalanced across participants (Fig. 1c). Half of the participants first performed the LARV blocks and then the LVRA blocks, the other half of the subjects vice versa. Before each block, an additional 10 min practice session was applied to get every participant adequately familiar with the hand-modality assignments and the experimental setup.

#### Experimental design

For the bimodal audiovisual targets, bimanual responses were required. We then classified the bimodal audiovisual trials into six different conditions according to the various types of participants’ performance^25–28^. Specifically, the bimodal trials with the preceding visual responses and the delayed auditory responses were classified as the Visual_Auditory (VA) trials; those with the preceding auditory responses and delayed visual responses were classified as the Auditory_Visual (AV) trials; those with the simultaneous visual and auditory responses were classified as the “Simultaneous” trials (i.e., the absolute RT difference between the visual and auditory responses should be less than 5 ms because of the uncertainty errors (2–5 ms) generated by the Presentation Software, indicating that the visual and auditory responses were made almost at the same time); those with only the visual responses were classified as the Visual_Only trials; those with only the auditory responses were classified as the Auditory_Only trials; and those without any responses were classified as the “Missed” trials. The VA and AV bimodal trials were the two bimodal conditions we were most interested in since those revealed the dominance effect of one particular sensory modality over the other one. Therefore, for the bimodal RT data, a 2 (sensory dominance: VA vs. AV trials) by 2 (response order: first vs. second response) by 2 (response hand: dominant vs. non-dominant hand) within-subject design was adopted.

### Statistical analysis

Omissions (3% and 0.5% for the unimodal and bimodal conditions, respectively), incorrect responses (4% and 9%), and outlier trials with RTs exceeding three SDs larger/smaller than the mean RT (1% and 1%) were excluded from further analysis. Subsequently, RTs in the correctly-responded bimodal trials were entered into a 2 (sensory dominance: VA vs. AV trials) by 2 (response order: first vs. second response) by 2 (response hand: dominant vs. non-dominant hand) within-subject repeated-measures ANOVA. Significant interactions were further interpreted by examining simple effects via *t* tests.

### Operational definition of the sensory dominance effect in bimodal trials

Three behavioral indexes in the bimodal trials were adopted to verify visual dominance over audition in the present experiment.

#### The size of the sensory dominance effect in bimodal VA vs. AV trials

The sequential order of the visual and auditory responses in the VA and AV trials indicated the direction of sensory dominance: vision was dominated by audition in the AV trials, and audition was dominated by vision in the VA trials. By calculating the RT difference between the second and the first response (RTsecond > RTfirst) for the VA and AV trials, respectively, we measured how much the first response preceded the second response in a given trial type. If the visual dominance effect in the VA trials was larger than the auditory dominance effect in the AV trials, we expected to observe a more considerable RT difference between the second and the first response in the VA than AV trials.

#### The psychological refractory period effect in the visual vs. auditory modality

Please note, since the first sensory dominance index above always utilizes the RT difference between the different sensory modalities, a confound of modality difference might be introduced. For example, if the unimodal visual responses are faster than the unimodal auditory responses, the RT difference between the second and the first response in the VA trials would be augmented. Likewise, the RT difference between the second and the first response in the AV trials would be diminished. Consequently, a more significanct sensory dominance effect in the VA than AV trials could be potentially confounded by modality difference effects.

Therefore, a complementary way to measure the sensory dominance effect is to compare the same sensory modality responses when the given modality wins vs. loses the multisensory competition. When individuals perform two sensorimotor tasks in immediate succession, the second task’s response is often delayed. This delay is called the psychological refractory period (PRP)^40,41^. In the present bimodal trials, the RT difference between responses of the same modality when it falls behind the other modality vs. when it precedes the other modality measures how much time it takes for this modality to recover from the PRP effect caused by preceding responses in the other modality when this modality loses the multisensory competition. For example, the difference in RTs to the visual components in the AV vs. VA trials measures how long it takes for the visual responses to recover from the PRP effect caused by the preceding auditory responses. Likewise, the difference in RTs to the auditory components in the VA vs. AV trials measures how long it takes for the auditory responses to recover from the PRP effect caused by the preceding visual responses. A significant visual dominance effect will manifest as a shorter time for the visual responses to recover from the PRP effect caused by the preceding auditory responses than vice versa. Since the PRP analyses results also revealed a sensory dominance effect for vision, we put the PRP results as the Supplementary Materials (see Supplementary Results S1 and Supplementary Fig. S1 online).

#### The proportion of VA vs. AV trials in the bimodal condition

In addition to the bimodal RT data, the visual dominance effect could be defined by larger proportions of VA than AV trials, indicating that vision wins the multisensory competition more frequently than audition.

### Categorization of subjects based on the dominant-hand-for-vision index in the unimodal trials

**Figure 2:**
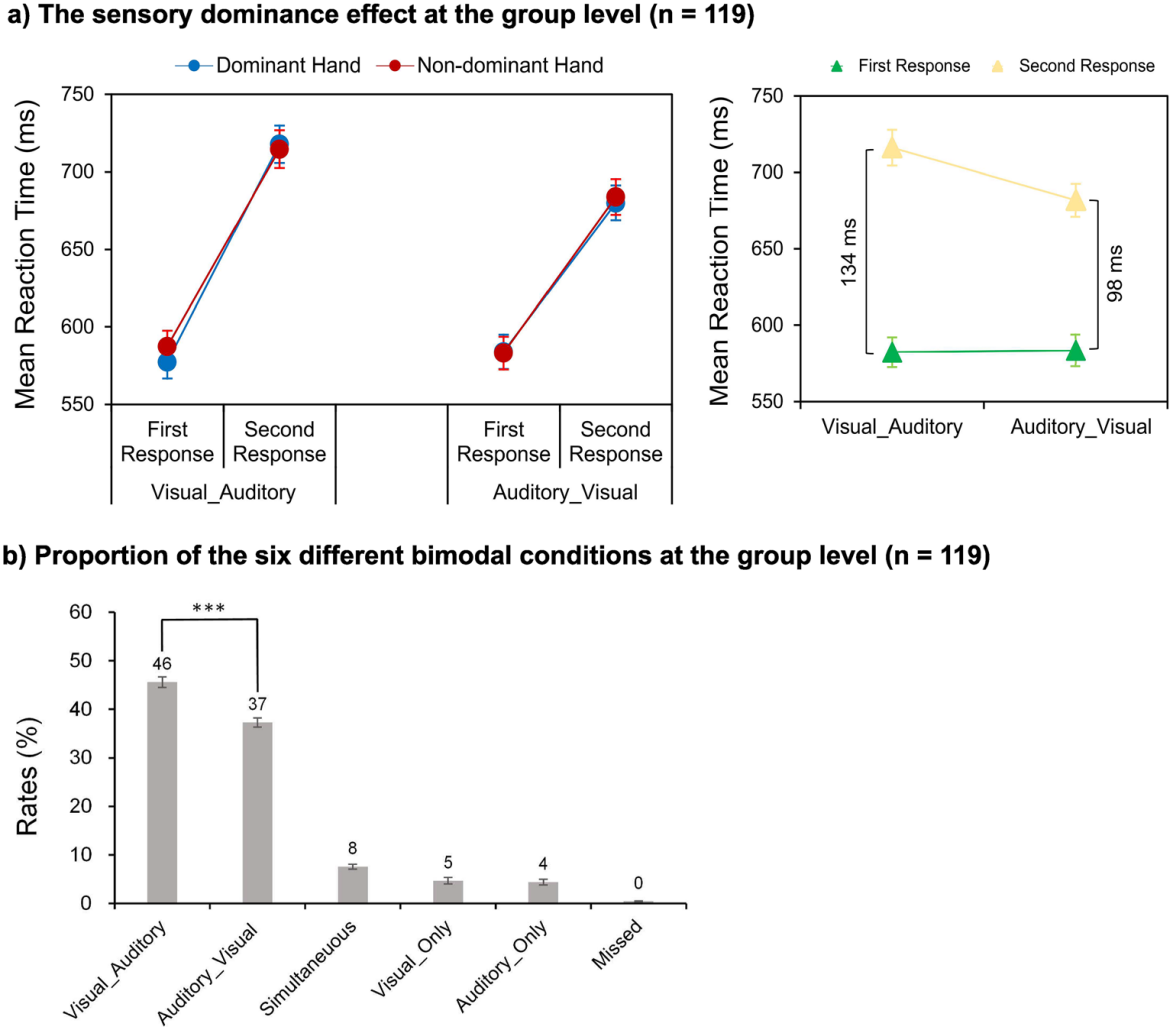
Robust visual dominance effects in the bimodal trials at the group level, n = 119. (**a**) Left panel, Mean RTs of the visual and the auditory components of the bimodal trials are shown as a function of the sensory dominance (VA vs. AV trials), the response order (first vs. second response), and the response hand (dominant vs. non-dominant hand). Right panel, The simple effects of the size of sensory dominance effect for the significant sensory dominance*response order interaction. (**b**) Proportion of the six different types of conditions in the bimodal trials. ***p < 0.001, **p < 0.01, *p < 0.05. The error bars show standard errors (SEs).

An intriguing result in the present study, based on bimodal RTs in the whole cohort of 119 right-handed subjects, was that no significant dominant hand benefits were found in these right-handers, i.e., the dominant (right) and non-dominant (left) hand responses were comparable (Fig. 2a, left and see Supplementary Fig. S1 online). This null dominant hand effect in the right-handers in the current crossmodal paradigm leads us to develop two hypotheses regarding the dominant vs. non-dominant preference for visual vs. auditory processing in humans. First, at the between-subject level, each individual is hypothesized to have their own dominant vs. non-dominant hand preference for the visual vs. auditory modality. Some individuals may prefer to use the dominant hand for visual responses and the non-dominant hand for auditory responses, and vice versa for the other individuals. Such individual differences would result in a null dominant hand effect when the data were collapsed over all these individuals with different hand preferences for different sensory modalities. Second, at the within-subject level, this preference for a given hand-modality association should remain stable across the unimodal and the bimodal conditions for each individual.

To test these two hypotheses, we first calculated the dominant-hand-for-vision index in the unimodal and the bimodal conditions for each participant. This index was defined as the difference between the size of the dominant hand effect (Non-dominant hand RT > Dominant hand RT) in the visual vs. auditory modality, i.e., [Visual RT (Non-dominant hand > Dominant hand)] − [Auditory RT (Non-dominant hand > Dominant hand). Thus, the dominant-hand-for-vision index included the RTs for both sensory modalities (vision, audition) and both hands (dominant and non-dominant hand). For the unimodal trials, the dominant and non-dominant hand RTs to the unimodal visual and auditory stimuli were used to calculate this dominant-hand-for-vision index. For the bimodal trials, the VA and AV trials were first collapsed, and then the dominant and non-dominant hand RTs to the visual and auditory components of the bimodal trials were used to calculate the dominant-hand-for-vision index in the bimodal condition. In this way, a positive value of this index indicates that the size of the dominant hand effect is larger for the visual than auditory stimuli, i.e., a dominant hand preference for vision. In contrast, a negative value indicates that the size of the dominant hand was larger for the auditory than visual stimuli, i.e., a dominant hand preference for audition. Subsequently, the Pearson correlation between the dominant-hand-for-vision index in the unimodal and the bimodal conditions was calculated. We predicted that if the hand-modality preference property stays stable within each individual, it should manifest in both the unimodal and the bimodal conditions, which are entirely independent, and correspondingly the dominant-hand-for-vision index in the unimodal and the bimodal condition should be significantly positively correlated.

**Figure 3:**
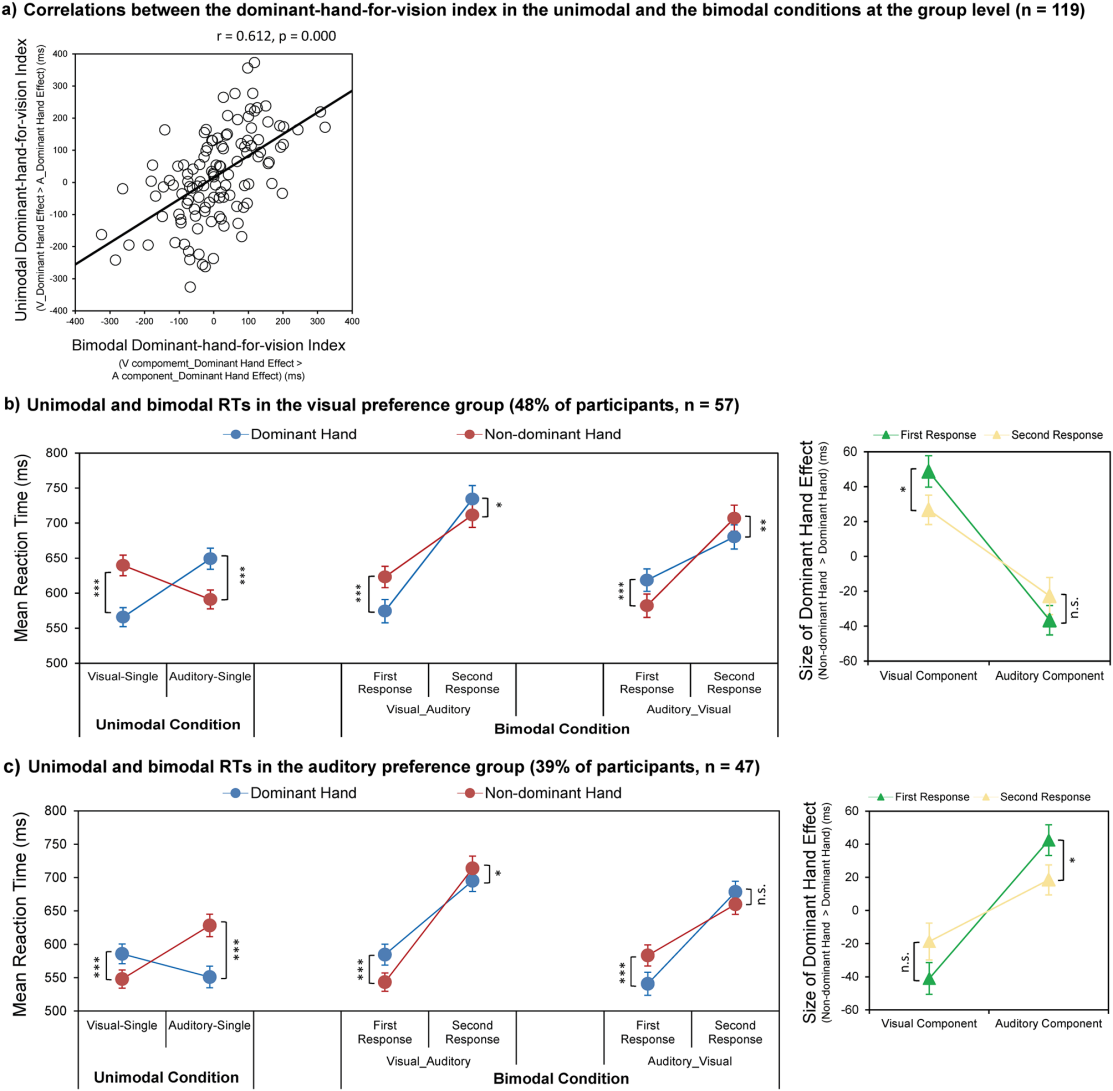
Two sub-groups with the opposite and stable preferential link between the visual vs. auditory modality and the dominant vs. non-dominant hand. (**a**) Correlations between the dominant-hand-for-vision index in the unimodal and the bimodal conditions for the whole group. (**b**) and (**c**) represented the unimodal and bimodal RT results for the visual preference group and the auditory preference group, respectively: left part of the Left panel, RTs in the unimodal trials are shown as a function of the sensory modality (visual vs. auditory) and the response hand (dominant vs. non-dominant hand); right part of the Left panel, RTs to the visual and auditory components of the bimodal trials are shown as a function of the response order (first vs. second response) and the response hand (dominant vs. non-dominant hand) for the VA and AV trials; Right panel, The simple effects for the significant sensory dominance*response order*response hand interaction in the bimodal condition. The dominant hand effect size is shown as a function of the sensory modality (visual vs. auditory) and the response order (first vs. second response). ***p < 0.001, **p < 0.01, *p < 0.05. The error bars show standard errors (SEs).

The correlation results verified our predictions and showed that roughly half of the subjects manifested a dominant hand preference for vision while the other half manifested a dominant hand preference for audition (Fig. 3a). The latter pattern very well accounted for the lack of a relevant dominant hand effect at the whole group level. We next categorized the 119 participants into two sub-groups to test how the different hand-modality preferences affect the interaction between sensory dominance and hand dominance. To this end, we categorized the participants based on the dominant-hand-for-vision index in the unimodal trials and tested the potentially different patterns of interaction between sensory dominance and hand dominance in the entirely independent set of bimodal trials between the two sub-groups. Specifically, the subjects with a positive dominant-hand-for-vision index in the unimodal trials were grouped as the “dominant hand preference for vision” group. In contrast, the subjects with a negative value were grouped as the “dominant hand preference for audition” group. Please note, since all the participants are right-handers, based on their scores on the Edinburgh Handedness Inventory, a subject will be excluded if she/he did not show an apparent right-hand effect (i.e., faster right- than left-hand responses) in responding to the unimodal stimuli of both modalities. Specifically, to be considered a real/relevant right-hand effect in responding to either the unimodal visual or auditory stimuli required that the right-hand responses should be at least 20 ms faster than the left-hand responses. This strict 20 ms cutoff was chosen based on previous evidence showing that the index finger RT difference between the right and left hand was about 6–16 ms^42–44^. Following this exclusion rule, 15 right-handed participants (13% of all the participants), who did not demonstrate a right-hand effect larger than 20 ms in either sensory modality, were excluded from further analysis. For the remaining 104 participants, 57 were categorized into the visual preference group (48% of the 119 participants), and the remaining 47 subjects were categorized into the auditory preference group (39% of the 119 participants).

First, to further validate the hypothesis that the hand-modality preference remains stable between the unimodal and bimodal trials and was opposite between the two sub-groups, a 2 (sensory modality: visual vs. auditory) by 2 (response hand: dominant vs. non-dominant hand) repeated-measures ANOVA was performed for the unimodal RTs for the two sub-groups. For the bimodal trials, RTs to the visual and auditory components of the VA and the AV trials were submitted to a 2 (sensory dominance: VA vs. AV trials) by 2 (response order: first vs. second response) by 2 (response hand: dominant vs. non-dominant hand) repeated-measures ANOVA for the two sub-groups.

To test how the different hand-modality preferences interact with the size of sensory dominance effect in the bimodal condition (i.e., the RT difference between the second and the first responses in the VA vs. AV trials), a 2 (hand-modality assignment: LARV vs. LVRA) by 2 (sensory dominance: VA vs. AV trials) by 2 (response order: first vs. second response) repeated-measures ANOVA was performed on the bimodal RTs for the two sub-groups. Also, a supplementary analysis on the interaction between the hand-modality preference and the PRP effect was performed. To this end, the bimodal RTs were submitted to a 2 (hand-modality assignment: LARV vs. LVRA) by 2 (sensory modality: visual vs. auditory) by 2 (response order: first vs. second responses) repeated-measures ANOVA for the two sub-groups (see Supplementary Results S2 and Supplementary Fig. S2 online). Moreover, for the proportion data in the bimodal trials, proportions of the VA and AV trials were submitted to a 2 (hand-modality assignment: LARV vs. LVRA) by 2 (sensory dominance: VA vs. AV trials) repeated-measures ANOVA. Since no significant interaction between the hand-modality preference and the proportions of the VA and AV trials was found, these results are presented in the Supplementary Materials (see Supplementary Results S3 and Supplementary Fig. S3 online). For the significant interactions from the ANOVAs, simple effects were further tested via planned *t* tests.

## Results

### Robust visual dominance effects in the bimodal trials at the group level (n = 119)

#### RTs in the bimodal trials

To test the potential interaction between hand dominance and sensory dominance, the bimodal RTs were submitted to a 2 (sensory dominance: VA vs. AV trials) by 2 (response order: first vs. second response) by 2 (response hand: dominant vs. non-dominant hand) repeated- measures ANOVA. The main effect of the sensory dominance was significant, *F*_(1,118)_ = 26.07, *p* < 0.001, *η*_p_^2^ = 0.181, indicating that RTs in the AV trials (633 ± 10 ms) were significantly faster than RTs in the VA trials (649 ± 10 ms). Consistent with the definition of bimodal trials, the main effect of the response order was significant, *F*_(1,118)_ = 245.64, *p* < 0.001, *η*_p_^2^ = 0.675, with faster first responses (583 ± 10 ms) than the second responses (699 ± 11 ms). The two-way interaction between the sensory dominance and the response order was significant, *F*_(1,118)_ = 68.44, *p* < 0.001, *η*_p_^2^ = 0.367. Further planned *t* tests on simple effects showed that the RT difference between the second and the first response was significantly larger in the VA (RTa > RTv = 134 ms) than AV trials (RTv > RTa = 98 ms), *t*_(118)_ = 8.27, *p* < 0.001, *d* = 0.758, indicating that the size of visual dominance effect in the VA trials was significantly larger than the size of auditory dominance effect in the AV trials (Fig. 2a, right). However, the main effect of the response hand was not significant, *F*_(1,118)_ = 1.81, *p* = 0.181, *η*_p_^2^ = 0.015, suggesting that no significant RT difference was found for the dominant hand (640 ± 10 ms) as compared to the non-dominant hands (642 ± 10 ms) for the present whole group of 119 right handers (Fig. 2a, left). Neither the two-way interaction between the sensory dominance and the response hand, *F*_(1,118)_ = 0.12, *p* = 0.727, *η*_p_^2^ = 0.001, nor the interaction between the response order and the response hand, *F*_(1,118)_ = 0.47, *p* = 0.495, *η*_p_^2^ = 0.004, was significant. The three-way interaction was also not significant, *F*_(1,118)_ = 0.74, *p* = 0.392, *η*_p_^2^ = 0.006.

#### Proportions of VA vs. AV trials in the bimodal trials

Proportions of the six different bimodal conditions are shown in Fig. 2b. The proportion of the VA trials (46% ± 1%) was significantly higher than the proportion of the AV trials (37% ± 1%), *t*_(118)_ = 4.92, *p* < 0.001, *d* = 0.453, indicating that visual responses preceded auditory responses more frequently than vice versa.

Taken together, at the group level, we replicated previous findings by showing a robust visual dominance effect in terms of the size of the sensory dominance effect (Fig. 2a, right) and the proportion of the VA vs. AV trials (Fig. 2b). However, no significant interaction between hand dominance and sensory dominance was revealed at the whole group level. Moreover, even no significant dominant hand advantage was found (Fig. 2a, left), although all the 119 participants were right-handed according to their scores from the Edinburgh Handedness Inventory.

### Correlation between the dominant-hand-for-vision index in the unimodal and bimodal conditions at the group level (n = 119)

The absence of a consistent superiority of the dominant hand for the whole group supports our hypothesis concerning individuals’ different hand-modality preferences. Some individuals might have their dominant hand preference for vision, and non-dominant hand preference for audition, and vice versa for other individuals. Therefore, no hand effect was found when the data were collapsed over all individuals irrespective of their hand preferences for different sensory modalities. We next calculated the Pearson correlation between the dominant-hand-for-vision index in the unimodal and the bimodal conditions to test the individual hand-modality preference’s stability. The two measures were significantly correlated, *r* = 0.612, *p* < 0.001 (Fig. 3a). Thus, participants showed the same hand-modality preference in the unimodal and bimodal trials. Besides, about half of the subjects showed a dominant hand preference for the visual modality, while the other half showed a dominant hand preference for the auditory modality. This inter-individual pattern can explain the lack of dominant hand effect at the group level when all 119 subjects’ data were collapsed for analysis.

### Verification of the opposite tendency of hand-modality preference between the two sub-groups in both the unimodal and the bimodal conditions

#### *Visual Preference group (48% of participants, n* = *57)*

##### The unimodal condition

To verify the potential interaction between sensory dominance and hand dominance in the unimodal condition, RTs of the unimodal trials were analyzed with a 2 (sensory modality: visual vs. auditory) by 2 (response hand: dominant vs. non-dominant hand) repeated-measures ANOVA. The main effect of the sensory modality was significant, *F*_(1,56)_ = 9.01, *p* = 0.004, *η*_p_^2^ = 0.139, indicating that RTs in the Visual-Single trials (603 ± 14 ms) were significantly faster than RTs in the Auditory-Single trials (620 ± 14 ms). The main effect of the response hand was not significant, *F*_(1,56)_ = 3.19, *p* = 0.079, *η*_p_^2^ = 0.054. The interaction was significant, *F*_(1,56)_ = 134.50, *p* < 0.001, *η*_p_^2^ = 0.706. Further planned *t* tests on simple effects showed that, for the unimodal visual targets, RTs of the dominant hand (566 ± 13 ms) were significantly faster than RTs of the non-dominant hand (640 ± 15 ms), *t*_(56)_ = 13.29, *p* < 0.001, *d* = 1.761, indicating a significant dominant hand effect for the unimodal visual processing. In contrast, for the unimodal auditory targets, RTs of the non-dominant hand (591 ± 13 ms) were significantly faster than RTs of the dominant hand (649 ± 15 ms), *t*_(56)_ = 6.80, *p* < 0.001, *d* = 0.900, indicating a significant non-dominant hand effect for the unimodal auditory processing (Fig. 3b, left).

##### The bimodal condition

To verify the potential interaction between sensory dominance and hand dominance in the bimodal condition, RTs to the visual and auditory components of the bimodal trials were submitted to a 2 (sensory dominance: VA vs. AV trials) by 2 (response order: first vs. second responses) by 2 (response hand: dominant vs. non-dominant hand) repeated-measures ANOVA. The main effect of the sensory dominance was significant, *F*_(1,56)_ = 10.28, *p* = 0.002, *η*_p_^2^ = 0.155, indicating that RTs in the AV trials (647 ± 16 ms) were significantly faster than RTs in the VA trials (661 ± 16 ms). In line with the definition of bimodal trials, the main effect of the response order was significant, *F*_(1,56)_ = 106.30, *p* < 0.001, *η*_p_^2^ = 0.655, with faster first (600 ± 15 ms) than second responses (708 ± 17 ms). The main effect of the response hand was not significant, *F*_(1,56)_ = 2.50, *p* = 0. 120, *η*_p_^2^ = 0.043. The two-way interaction between the sensory dominance and the response order, *F*_(1,56)_ = 37.13, *p* < 0.001, *η*_p_^2^ = 0.399, and the interaction between the sensory dominance and the response hand, *F*_(1,56)_ = 5.83, *p* = 0.019, *η*_p_^2^ = 0.094, were both significant. The two-way interaction between the response order and the response hand was not significant, *F*_(1,56)_ = 0.19, *p* = 0.665, *η*_p_^2^ = 0.003. However, the three-way interaction was significant, *F*_(1,56)_ = 27.16, *p* < 0.001, *η*_p_^2^ = 0.327.

To further explain the three-way interaction, we separately computed two repeated-measures ANOVA with factors of the response order (first vs. second response) and the response hand (dominant vs. non-dominant hand) for the VA and AV bimodal trials. For the VA trials (Fig. 3b, left), the main effect of the response order, *F*_(1,56)_ = 110.89, *p* < 0.001, *η*_p_^2^ = 0.664, and the main effect of the response hand, *F*_(1,56)_ = 5.81, *p* = 0.019, *η*_p_^2^ = 0.094, were both significant. The interaction was also significant, *F*_(1,56)_ = 18.80, *p* < 0.001, *η*_p_^2^ = 0.251. Further planned *t* tests on simple effects revealed that, for the first visual responses in the VA trials, RTs of the dominant hand (574 ± 17 ms) were significantly faster than RTs of the non-dominant hand (623 ± 15 ms), *t*_(56)_ = 5.43, *p* < 0.001, *d* = 0.720, indicating a significant dominant hand effect for the winning visual responses of the bimodal trials. In contrast, for the second auditory responses, RTs of the non-dominant hand (712 ± 18 ms) were significantly faster than RTs of the dominant hand (734 ± 19 ms), *t*_(56)_ = 2.13, *p* = 0.038, *d* = 0.282, indicating a significant non-dominant hand effect for the losing auditory responses. In terms of the AV trials (Fig. 3b, left), the analysis revealed a significant main effect of the response order, *F*_(1,56)_ = 90.50, *p* < 0.001, *η*_p_^2^ = 0.618. The main effect of the response hand was not significant, *F*_(1,56) =_ 2.09, *p* = 0.154, *η*_p_^2^ = 0.036. The interaction was significant, *F*_(1,56)_ = 16.88, *p* < 0.001, *η*_p_^2^ = 0.232. Further planned *t* tests on simple effects showed that, for the first auditory responses in the AV trials, RTs of the non-dominant hand (582 ± 17 ms) were significantly faster than RTs of the dominant hand (619 ± 16 ms), *t*_(56)_ = 4.34, *p* < 0.001, *d* = 0.575, indicating a significant non-dominant hand effect for the winning auditory responses of the bimodal trials. In contrast, for the second visual responses, RTs of the dominant hand (680 ± 17 ms) were faster than RTs of the non-dominant hand (707 ± 19 ms), *t*_(56)_ = 3.18, *p* = 0.002, *d* = 0.421, indicating a significant dominant hand effect for the losing visual responses. Therefore, irrespective of the direction of sensory dominance, i.e., in both the visual dominance (VA) and the auditory dominance (AV) trials, the visual preference group showed a significant dominant hand effect manifested in responding to the visual components of the bimodal stimuli and a significant non-dominant hand effect in responding to the auditory components. Moreover, the size of the dominant hand effect in responding to the visual components of the bimodal trials was significantly larger when vision dominated audition (i.e., in the VA trials, 49 ± 9 ms) than when audition dominated vision (i.e., in the AV trials, 27 ± 8 ms), *t*_(56)_ = 2.13, *p* = 0.038, *d* = 0.282. For the size of the non-dominant hand effect in responding to the auditory components, however, no significant difference was found between the AV (37 ± 8 ms) and VA (23 ± 11 ms) trials, *t*_(56)_ = 1.04, *p* = 0.303, *d* = 0.138 (Fig. 3b, right). For the visual preference group, these results suggested that the dominant hand effect was boosted when vision was associated with the dominant hand and when vision won the multisensory competition.

#### *Auditory Preference group (39% of participants, n* = *47)*

##### The unimodal condition

To verify the potential interaction between sensory dominance and hand dominance in the unimodal condition, RTs of the unimodal trials were submitted into a 2 (sensory modality: visual vs. auditory) by 2 (response hand: dominant vs. non-dominant hand) repeated-measures ANOVA. The main effect of the sensory modality was significant, *F*_(1,46)_ = 11.57, *p* = 0.001, *η*_p_^2^ = 0.201, indicating that RTs in the Visual-Single trials (567 ± 14 ms) were significantly faster than RTs in the Auditory-Single trials (590 ± 16 ms). The main effect of the response hand was significant, *F*_(1,46)_ = 29.67, *p* < 0.001, *η*_p_^2^ = 0.392, suggesting that RTs of the dominant hand (568 ± 15 ms) were significantly faster than RTs of the non-dominant hand (588 ± 15 ms). The interaction was also significant, *F*_(1,46)_ = 93.62, *p* < 0.001, *η*_p_^2^ = 0.671. Further planned *t* tests on simple effects showed that for the unimodal visual targets, RTs of the non-dominant hand (548 ± 14 ms) were significantly faster than RTs of the dominant hand (586 ± 15 ms), *t*_(46)_ = 4.95, *p* < 0.001, *d* = 0.722, suggesting a significant non-dominant hand effect for the unimodal visual processing. In contrast, for the unimodal auditory targets, RTs of the dominant hand (551 ± 16 ms) were significantly faster than RTs of the non-dominant hand (628 ± 17 ms), *t*_(46)_ = 12.42, *p* < 0.001, *d* = 1.812, suggesting a significant dominant hand effect for the unimodal auditory processing (Fig. 3c, left).

##### The bimodal condition

To verify the potential interaction between sensory dominance and hand dominance in the bimodal condition, RTs to the visual and auditory components of the bimodal trials were submitted into a 2 (sensory dominance: VA vs. AV trials) by 2 (response order: first vs. second responses) by 2 (response hand: dominant vs. non-dominant hand) repeated measures ANOVA. The main effect of the sensory dominance was significant, *F*_(1,46)_ = 10.76, *p* = 0.002, *η*_p_^2^ = 0.190, indicating that RTs in the AV trials (616 ± 14 ms) were significantly faster than RTs in the VA trials (634 ± 14 ms). In line with the definition of bimodal trials, the main effect of the response order was significant, *F*_(1,46)_ = 99.73, *p* < 0.001, *η*_p_^2^ = 0.684, with faster first responses (563 ± 15 ms) than the second responses (687 ± 15 ms). The main effect of the response hand was not significant, *F*_(1,46)_ = 0.01, *p* = 0.924, *η*_p_^2^ = 0.000. The two-way interaction between the sensory dominance and the response order, *F*_(1,46)_ = 25.58, *p* < 0.001, *η*_p_^2^ = 0.357, and the interaction between the sensory dominance and the response hand, *F*_(1,46)_ = 8.56, *p* = 0.005, *η*_p_^2^ = 0.157, were both significant. The two-way interaction between the response order and the response hand was not significant, *F*_(1,46)_ = 0.01, *p* = 0.934, *η*_p_^2^ = 0.000. Three-way interaction was significant, *F*_(1,46)_ = 18.96, *p* < 0.001, *η*_p_^2^ = 0.292.

To further indicate the three-way interaction, two repeated-measures ANOVA with the factors response order (first vs. second response) and response hand (dominant vs. non-dominant hand) were applied for the VA and AV bimodal trials. For the VA trials (Fig. 3c, left), the main effect of the response order, *F*_(1,46)_ = 105.22, *p* < 0.001, *η*_p_^2^ = 0.696, and the main effect of the response hand, *F*_(1,46)_ = 4.78, *p* = 0.034, *η*_p_^2^ = 0.094, were both significant. The interaction reached significance as well, *F*_(1,46)_ = 14.59, *p* < 0.001, *η*_p_^2^ = 0.241. Further planned *t* tests on simple effects showed that for the first visual responses in the VA trials, RTs of the non-dominant hand (543 ± 14 ms) were significantly faster than RTs of the dominant hand (584 ± 16 ms), *t*_(46)_ = 4.27, *p* < 0.001, *d* = 0.623, indicating a significant non-dominant hand effect for the winning visual responses of the bimodal trials. In contrast, for the second auditory response, RTs of the dominant hand (695 ± 16 ms) were significantly faster than RTs of the non-dominant hand (714 ± 18 ms), *t*_(46)_ = 2.04, *p* = 0.047, *d* = 0.297, indicating a significant dominant hand effect for the losing auditory responses. For the AV trials (Fig. 3c, left), the main effect of the response order, *F*_(1,46)_ = 81.05, *p* < 0.001, *η*_p_^2^ = 0.638, and the main effect of the response hand, *F*_(1,46)_ = 6.43, *p* = 0.015, *η*_p_^2^ = 0.123, were both significant. The interaction was also significant, *F*_(1,46)_ = 11.20, *p* = 0.002, *η*_p_^2^ = 0.196. Further planned *t* tests on simple effects revealed that for the first auditory response in the AV trials, RTs of the dominant hand (541 ± 17 ms) were significantly faster than RTs of the non-dominant hand (583 ± 16 ms), *t*_(46)_ = 4.55, *p* < 0.001, *d* = 0.664. This finding indicates a significant dominant hand effect for the winning auditory responses. In contrast, for the second visual response, clear numerically faster non-dominant (660 ± 15 ms) than dominant (679 ± 16 ms) hand responses were observed, but the RT difference did not reach statistical significance, *t*_(46)_ = 1.68, *p* = 0.099, *d* = 0.246. Therefore, both the VA and AV bimodal trials suggested a significant dominant hand effect in responding to the auditory components, regardless of the direction of sensory dominance. By contrast, the non-dominant hand effect was only observed for the winning visual components in the VA trials but was eliminated for the losing visual components in the AV trials. Moreover, the size of the dominant hand effect for the auditory components of the bimodal trials was significantly larger when audition dominated vision (i.e., in the AV trials, 42 ± 9 ms) than when vision dominated audition (i.e., in the VA trials, 19 ± 9 ms), *t*_(46)_ = 2.07, *p* = 0.044, *d* = 0.302. In contrast, for the size of the non-dominant hand effect manifested in responding to the visual components, no significant difference was found between the VA and (41 ± 10 ms) and AV (19 ± 11 ms) trials, *t*_(46)_ = 1.64, *p* = 0.107, *d* = 0.240 (Fig. 3c, right). Therefore, for the auditory preference group, the hand effect was boosted when audition was associated with the dominant hand and won the multisensory competition.

Taken together, consistent with our hypothesis, both unimodal and bimodal trials suggested that there were two sub-groups with opposite hand-modality preference: the visual preference group (n = 57) showed a dominant hand preference for the visual stimuli and a non-dominant hand preference for the auditory stimuli (Fig. 3b, left). In contrast, the auditory preference group (n = 47) showed a dominant hand preference for auditory stimuli and a non-dominant hand preference for visual stimuli (Fig. 3c, left). More intriguing, both groups showed stability in hand-modality preference irrespective of the direction of sensory dominance during multisensory competition (i.e., in both the VA and AV bimodal trials). Besides, the dominant hand effect was enhanced when the preferred sensory modality was paired with the dominant hand (i.e., when subjects belonging to the visual preference group used their dominant hand for visual responses, and subjects belonging to the auditory preference group used their dominant hand for auditory responses), and when this modality won the multisensory competition (Fig. 3b, c, right).

### Interaction between the hand-modality preference and the size of sensory dominance effect (sub-group data)

#### *Visual preference group (48% of participants, n* = *57)*

**Figure 4:**
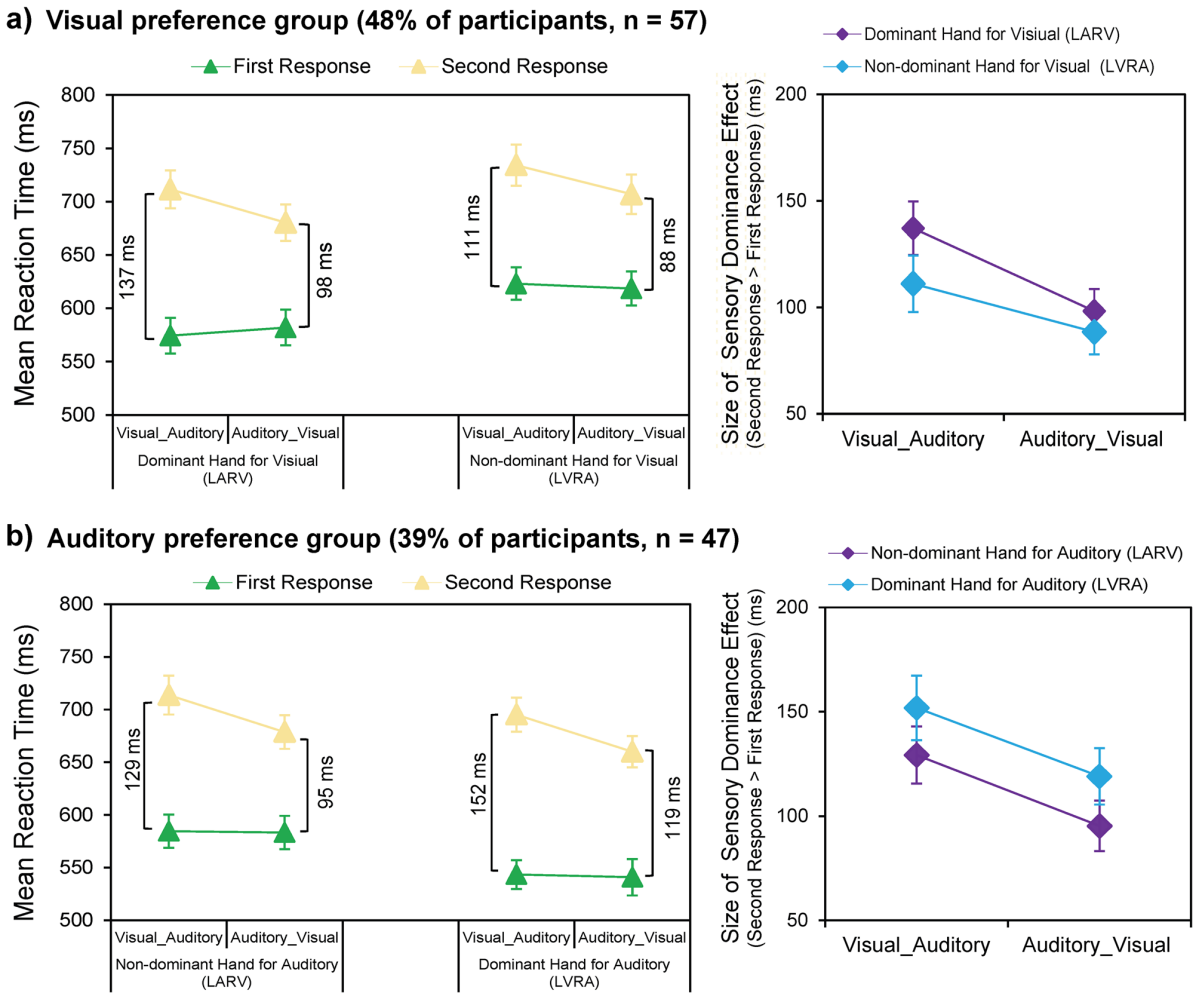
Interaction between the hand-modality preference and the sensory dominance for the *visual* preference group (**a**) and the *auditory* preference group (**b**). Left panel, Bimodal RTs are shown as a function of the sensory dominance (VA vs. AV trials) and the response order (first vs. second response) for the LARV (left part) and the LVRA (right part) task. Right panel, The simple effects for the significant sensory dominance*response order interaction. The sizes of the sensory dominance effects for the VA (RTa > RTv) and AV (RTv > RTa) trials are shown as a function of hand-modality assignment (LARV vs. LVRA). The error bars show standard errors (SEs).

RTs to the visual and auditory components of the bimodal trials were submitted to a 2 (hand-modality assignment: LARV vs. LVRA) by 2 (sensory dominance: VA vs. AV trials) by 2 (response order: first vs. second response) repeated-measures ANOVA to test how the hand-modality preference modulated the size of sensory dominance. The main effect of the hand-modality assignment was significant, *F*_(1,56)_ = 27.16, *p* < 0.001, *η*_p_^2^ = 0.327, indicating that bimodal RTs were significantly faster when the dominant hand was assigned to the visual target (i.e., in the LARV condition, 637 ± 16 ms) than when the dominant hand was assigned to the auditory target (i.e., in the LVRA condition, 671 ± 16 ms). The main effect of the sensory dominance was significant, *F*_(1,56)_ = 10.28, *p* = 0.002, *η*_p_^2^ = 0.155, indicating that RTs in the AV trials (647 ± 16 ms) were significantly faster than RTs in the VA trials (661 ± 16 ms). The main effect of the response order was significant, *F*_(1,56)_ = 106.30, *p* < 0.001, *η*_p_^2^ = 0.655. Moreover, the two-way interaction between the sensory dominance and the response order was significant, *F*_(1,56)_ = 37.13, *p* < 0.001, *η*_p_^2^ = 0.399. Further planned *t* tests on simple effects revealed that the size of sensory dominance effect was larger in the VA trials (RTa > RTv = 124 ± 12 ms) than in the AV trials (RTv > RTa = 93 ± 10 ms), *t*_(56)_ = 6.09, *p* < 0.001, *d* = 0.807 (Fig. 4a, right). Thus, irrespective of which sensory modality was mapped to the dominant hand, i.e., in both the LARV and LVRA conditions, the visual dominance effect in the VA trials was significantly larger than the auditory dominance effect in the AV trials. Also, the two-way interaction between the hand-modality assignment and the response order reached significance, *F*_(1,56)_ = 5.83, *p* = 0.019, *η*_p_^2^ = 0.094. Further planned *t* tests on simple effects suggested that the size of sensory dominance effect in the LARV condition (RTsecond > RTfirst = 118 ± 11 ms) was significantly larger than in the LVRA condition (RTsecond > RTfirst = 100 ± 11 ms), *t*_(56)_ = 2.42, *p* = 0.019, *d* = 0.320 (Fig. 4a, right). This result indicated that, regardless of the direction of the sensory dominance, i.e., for both the visual dominance effect (VA trials) and the auditory dominance effect (AV trials), the sensory dominance effect was boosted when the visual target was paired with the preferred dominant hand (i.e., in the LARV condition) for the visual preference group. Neither the two-way interaction between the hand-modality assignment and the sensory dominance, *F*_(1,56)_ = 0.19, *p* = 0.665, *η*_p_^2^ = 0.003, nor the three-way interaction, *F*_(1,56)_ = 2.50, *p* = 0.120, *η*_p_^2^ = 0.043, was significant.

#### *Auditory preference group (39% of participants, n* = *47)*

RTs to the visual and auditory components of the bimodal trials were submitted to a 2 (hand-modality assignment: LARV vs. LVRA) by 2 (sensory dominance: VA vs. AV trials) by 2 (response order: first vs. second responses) repeated-measures ANOVA to test how the hand-modality preference modulated the size of sensory dominance. The main effect of the hand-modality assignment was significant, *F*_(1,46) =_ 18.96, *p* < 0.001, *η*_p_^2^ = 0.292, indicating that bimodal RTs were significantly faster when the dominant hand was assigned to the auditory target (i.e., in the LVRA condition, 610 ± 13 ms) than when the dominant hand was assigned to the visual target (i.e., in the LARV condition 640 ± 15 ms). The main effect of the sensory dominance was significant, *F*_(1,46)_ = 10.76, *p* = 0.002, *η*_p_^2^ = 0.190, indicating that RTs in the AV trials (616 ± 14 ms) were significantly faster than RTs in the VA trials (634 ± 14 ms). The main effect of the response order was significant too, *F*_(1,46)_ = 99.73, *p* < 0.001, *η*_p_^2^ = 0.684. Moreover, the two-way interaction between the sensory dominance and the response order was significant, *F*_(1,46)_ = 25.58, *p* < 0.001, *η*_p_^2^ = 0.357. Further planned *t* tests on simple effects indicated that, irrespective of which sensory modality was assigned to the dominant hand, i.e., both in the LARV and LVRA conditions, the size of visual dominance effect in the VA trials (RTa > RTv = 141 ± 14 ms) was significantly larger than the size of auditory dominance effect in the AV trials (RTv > RTa = 107 ± 12 ms), *t*_(46)_ = 5.06, *p* < 0.001, *d* = 0.738 (Fig. 4b, right). In addition, the two-way interaction between the hand-modality assignment and the response order also reached significance, *F*_(1,46)_ = 8.56, *p* = 0.005, *η*_p_^2^ = 0.157. Further planned *t* tests on simple effects revealed that the size of sensory dominance effect in the bimodal condition was significant larger in the LVRA task (RTsecond > RTfirst = 135 ± 14 ms) than in the LARV task (RTsecond > RTfirst = 112 ± 12 ms), *t*_(46)_ = 2.93, *p* = 0.005, *d* = 0.427 (Fig. 4b, right). These results thus indicated that regardless of the direction of sensory dominance, i.e., for both the visual dominance effect (VA trials) and the auditory dominance effect (AV trials), the sensory dominance effect was boosted when the auditory target was paired with the dominant hand (i.e., in the LVRA condition) for the auditory preference group. Neither the two-way interaction between the hand-modality assignment and the sensory dominance, *F*_(1,46)_ = 0.07, *p* = 0.934, *η*_p_^2^ = 0.000, nor the three-way interaction, *F*_(1,46)_ = 0.09, *p* = 0.924, *η*_p_^2^ = 0.000, was significant.

Taken together, both groups showed a robust visual dominance effect in terms of the size of sensory dominance. Regardless of which sensory modality was assigned to the dominant hand, i.e., in both the LARV and LVRA conditions, the size of the visual dominance effect (VA trials) was significantly larger than the size of the auditory dominance effect (AV trials) (Fig. 4a, b, right). The visual dominance effect could not be reversed or eliminated by using the non-dominant hand when responding to visual stimuli. Moreover, when the hand-modality assignment was congruent with the specific hand-modality preference of each group (i.e., in the LARV condition for the visual preference group and in the LVRA condition for the auditory preference group), not only participants’ reaction to the bimodal trials was faster (Fig. 4a, b, left), but also the sensory dominance effect was boosted (Fig. 4a, b, right). This indicates that the individual hand-modality preference could modulate the size of the sensory dominance effect.

## Discussion

In the present behavioral study, we aimed at investigating the potential interaction between the sensory and the hand dominance effect in a large group of right-handed participants (n = 119). For the whole group of participants, we replicated previous findings by showing a robust visual dominance effect in three separate analyses: First, the frequency of trials in which the visual responses preceded the auditory responses (Visual_Auditory (VA) trials) was significantly higher than the frequency of the inverse trials (Auditory_Visual (AV) trials) (Fig. 2b). Second, the size of the visual dominance effect in the Visual_Auditory trials was significantly larger than the size of the auditory dominance effect in the Auditory_Visual trials (Fig. 2a, right). Third, even when the visual responses were preceded (dominated) by auditory responses, it took a shorter time for the visual responses to recover from the psychological refractory period (PRP) caused by the previous auditory responses than vice versa (see Supplementary Results S1 and Supplementary Fig. S1 online)^25,26,28^.

### Individual difference in the hand-modality preferential link across human right-handers

The present study’s most novel and intriguing finding is that right-handers showed an individual and persistent preferential link between a given sensory modality (visual vs. auditory) and their dominant vs. non-dominant hand. Notably, this preferential link remained stable across independent groups (unimodal and bimodal) of trials (Fig. 3a). Specifically, most of the right-handed participants could be categorized into two sub-groups, each with a characteristic preferential link between the visual vs. auditory modality and the dominant vs. non-dominant hand: 57 participants (i.e., 48% of the current 119 right-handed subjects) showed a stable dominant hand preference for vision and a non-dominant hand preference for audition (i.e., the visual preference (VP) group) (Fig. 3b). In contrast, 47 (i.e., 39%) of the right-handed subjects constituted the auditory preference (AP) group, i.e., they showed a stable dominant hand preference for auditory stimuli (Fig. 3c).

Previous findings regarding the reaction time (RT) advantage of the dominant hand in response to the visual and auditory stimuli were inconsistent^23,29–32^. The present results suggest that this inconsistency is likely due to a sizeable inter-individual variance in the hand-modality preference. The group-averaged dominant hand effect (i.e., the RT difference between the dominant and non-dominant hand) could be either present, eliminated, or reversed, depending on the proportion of the visual preference vs. auditory preference subjects in the recruited sample in various studies. An even mix of the visual preference and the auditory preference subjects will result in a null dominant hand effect, while a significant unbalanced proportion towards one type of subjects will result in either a significant dominant hand effect or a reversed effect.

To the best of our knowledge, there are two seminal studies on the potential individual difference in the responding speed of the dominant right hand to the visual vs. auditory stimuli^36,37^. In a more complex object discrimination task, subjects were asked to use two fingers of their right hand to indicate which of two predefined visual features (i.e., a horizontal vs. vertical ellipse) was presented in the visual condition and which of two predefined tones (i.e., 540 Hz vs. 560 Hz) was present in the auditory condition^37^. The results showed that the subjects (n = 24) could be categorized as either “auditory dominant” or “visual dominant”, based on whether their dominant right hand responded faster to one or the other modality. 14 of 24 subjects (58%) showed shorter RTs for the unimodal visual targets. In comparison, 10 subjects (42%) performed better for the unimodal auditory targets. In the present study, a detection task was adopted on simple unimodal visual (perceptual contrast) and auditory (pure tone) features, and not only the dominant but also the non-dominant hand responses were recorded for both the visual and the auditory modality in a large cohort (n = 119) of subjects. Our results further suggested that the dominant hand preference for one sensory modality is accompanied by a non-dominant hand preference for the other modality. Specifically speaking, the dominant hand responded better to the visual targets for the visual preference group while the non-dominant hand responded better to the auditory targets and vice versa for the auditory preference group (Fig. 3b, c, left). With previous evidence, the present results showed each human right-hander possesses a stable preferential hand-modality link, irrespective of experimental tasks and complexity of stimulus features, and this preferential link varies across individuals.

### Mutual boosting of the sensory dominance effect and the hand dominance effect

The present results further suggested that both the visual preference and the auditory preference groups showed a consistent pattern of mutual interaction between the sensory and the hand dominance effect. First, the hand dominance effect was boosted by the sensory dominance effect. Whenever the dominant hand was paired with its preferred modality and won the multisensory competition (i.e., when vision won the multisensory competition in the right hand-vision correspondence condition for the visual preference group, and when audition won multisensory competition in the right hand-audition correspondence condition for the auditory preference group), the hand dominance effect was boosted (Fig. 3b, c, right). Second, the sensory dominance effect was boosted by the preferred hand-modality preference. Both the size of the sensory dominance effect (Fig. 4a, b, right) and the PRP effect (see Supplementary Results S2 and Supplementary Fig. S2 online) were boosted when the visual and the auditory modalities were paired with their preferred hand, such as in the ‘LARV’ condition for the visual preference group and the ‘LVRA’ condition for the auditory preference group.

It has been suggested that the sensory systems dynamically interact with the prefrontal cortex, the sensorimotor cortex, and the default mode network (DMN) during multisensory competition: the “winning” sensory system is coupled with the prefrontal and the sensorimotor networks, while the “losing” sensory system is coupled with the default mode network^26,28^. Although the underlying neural mechanisms of the mutual boosting between the sensory and the hand dominance effect remain unclear, it is tentative to assume that when a sensory modality is paired with its preferred hand, as in the ‘LARV’ condition for the visual preference group and the ‘LVRA’ condition for the auditory preference group, the information flow from the sensory cortex to the corresponding sensorimotor representations is optimized^45^. The optimized information flow from the sensory cortex to the higher-order neural networks further increases the neural coupling between the “winning” sensory system and the prefrontal and the sensorimotor networks, as well as the neural coupling between the “losing” sensory system and the DMN. Accordingly, the sensory dominance effect of the “winning” modality is boosted due to the increased neural coupling between the “winning” sensory system and the prefrontal and sensorimotor networks, and it takes even longer time for the “losing” modality to recover from the PRP effect caused by the “winning” modality due to the increased neural coupling between the “losing” sensory system and the DMN. Following the same logic, when the dominant hand is paired with its preferred modality, and the preferred modality wins the multisensory competition, the superior processing efficiency of the motor dominant over the (motor) non-dominant hemisphere is further enlarged, and accordingly a boosted dominant hand effect is induced.

To summarize, the current study provides novel evidence suggesting that each human right-hander shows a stable preferential hand-modality link. Although this preferential link stays consistent within an individual irrespective of experimental tasks and complexity of stimuli, it varies dramatically across individuals. Also, the individual difference in the hand-modality preference results in significant interactions between sensory dominance and the hand dominance effects, mutually boosting each other. Future brain imaging studies are warranted to explore the neural mechanisms underlying the above behavioral phenomena. Besides, it remains to be investigated whether a similar pattern of results is found in left-handed subjects.

## Supplementary information

Supplementary Information.

## Data Availability

The datasets generated during and/or analyzed during the current study are available from the corresponding author on reasonable request.

## References

[1] Driver J, Spence C (1998). Cross-modal links in spatial attention. Philos. Trans. R. Soc. Lond. Ser. B Biol. Sci..

[2] Driver J (2001). A selective review of selective attention research from the past century. Br. J. Psychol..

[3] Macaluso E, Frith CD, Driver J (2000). Modulation of human visual cortex by crosmodal spatial attention. Science (80-).

[4] Spence C, Parise C, Chen Y, Murray MM, Wallace MT (2012). The Colavita visual dominance effect. The Neural Bases of Multisensory Processes.

[5] Spence C, Srinivasan N (2009). Explaining the Colavita visual dominance effect. Progress in Brain Research.

[6] Robinson CW, Ahmar N, Sloutsky VM (2010). Evidence for auditory dominance in a passive oddball task. Proc. Annu. Meet. Cogn. Sci. Soc..

[7] Robinson CW, Chandra M, Sinnett S (2016). Existence of competing modality dominances. Atten. Percept. Psychophys..

[8] Spence C, Wixted JT (2018). Multisensory perception. Stevens’ Handbook of Experimental Psychology and Cognitive Neuroscience.

[9] Colavita FB (1974). Human sensory dominance. Atten. Percept. Psychophys..

[10] Welch RB, Warren DH (1980). Immediate perceptual response to intersensory discrepancy. Psychol. Bull..

[11] Galletti C, Fattori P (2018). The dorsal visual stream revisited: stable circuits or dynamic pathways?. Cortex.

[12] Rauschecker JP (2018). Where, when, and how: are they all sensorimotor? Towards a unified view of the dorsal pathway in vision and audition. Cortex.

[13] Chen JL, Penhune VB, Zatorre RJ (2009). The role of auditory and premotor cortex in sensorimotor transformations. Ann. N. Y. Acad. Sci..

[14] Rauschecker JP (2011). An expanded role for the dorsal auditory pathway in sensorimotor control and integration. Hear. Res..

[15] Johnson JA, Strafella AP, Zatorre RJ (2007). The role of the dorsolateral prefrontal cortex in bimodal divided attention: two transcranial magnetic stimulation studies. J. Cogn. Neurosci..

[16] Goble DJ, Brown SH (2008). The biological and behavioral basis of upper limb asymmetries in sensorimotor performance. Neurosci. Biobehav. Rev..

[17] Corballis MC (2003). From mouth to hand: gesture, speech, and the evolution of right-handedness. Behav. Brain Sci..

[18] Teixeira LA (2008). Categories of manual asymmetry and their variation with advancing age. Cortex.

[19] Verstynen T, Diedrichsen J, Albert N, Aparicio P, Ivry RB (2005). Ipsilateral motor cortex activity during unimanual hand movements relates to task complexity. J. Neurophysiol..

[20] Triggs WJ, Calvanio R, Levine M (1997). Transcranial magnetic stimulation reveals a hemispheric asymmetry correlate of intermanual differences in motor performance. Neuropsychologia.

[21] Todor JI, Kyprie PM, Price HL (1982). Lateral asymmetries in arm, wrist and finger movements. Cortex.

[22] Peters M, Durding B (1979). Left-handers and right-handers compared on a motor task. J. Mot. Behav..

[23] Annett M, Annett J (1979). Individual differences in right and left reaction time. Br. J. Psychol..

[24] Carson RG, Chua R, Goodman D, Byblow WD, Elliott D (1995). The preparation of aiming movements. Brain Cogn..

[25] Yue Z, Jiang Y, Li Y, Wang P, Chen Q (2015). Enhanced visual dominance in far space. Exp. Brain Res..

[26] Huang S (2015). Multisensory competition is modulated by sensory pathway interactions with fronto-sensorimotor and default-mode network regions. J. Neurosci..

[27] Fang Y, Li Y, Xu X, Tao H, Chen Q (2020). Top-down attention modulates the direction and magnitude of sensory dominance. Exp. Brain Res..

[28] Li Y (2017). Neurophysiological correlates of visual dominance: a lateralized readiness potential investigation. Front. Psychol..

[29] Chan AHS, Ng AWY (2012). Finger response times to visual, auditory and tactile modality stimuli. Lect. Notes Eng. Comput. Sci..

[30] Schröter H, Leuthold H (2008). Effects of response sequence length on motor programming: a chronometric analysis. Acta Psychol. (Amst).

[31] Kourtis D, Vingerhoets G (2016). Evidence for dissociable effects of handedness and consistency of hand preference in allocation of attention and movement planning: an EEG investigation. Neuropsychologia.

[32] Sathiamoorthy A, Sathiamoorthy SS, Bhat SK, Hiremath S, Shenoy N (1994). Influence of handedness on the visual and auditory reaction time. Indian J. Physiol. Pharmacol..

[33] Hiraoka K (2018). The laterality of stop and go processes of the motor response in left-handed and right-handed individuals. Laterality.

[34] Lofthus GK (1981). Sensorimotor performance and limb preference. Percept. Mot. Skills.

[35] Woodworth RS (1938). Experimental psychology.

[36] Fort A, Delpuech C, Pernier J, Giard MH (2002). Early auditory-visual interactions in human cortex during nonredundant target identification. Cogn. Brain Res..

[37] Giard MH, Peronnet F (1999). Auditory-visual integration during multimodal object recognition in humans: a behavioral and electrophysiological study. J. Cogn. Neurosci..

[38] Oldfield RC (1971). The assessment and analysis of handedness: the Edinburgh inventory. Neuropsychologia.

[39] Pool EM, Rehme AK, Fink GR, Eickhoff SB, Grefkes C (2014). Handedness and effective connectivity of the motor system. Neuroimage.

[40] Pashler H (1994). Dual-task interference in simple tasks: data and theory. Psychol. Bull..

[41] Telford CW (1931). The refractory phase of voluntary and associative responses. J. Exp. Psychol..

[42] Kerr M, Mingay R, Elithorn A (1963). Cerebral dominance in reaction time responses. Br. J. Psychol..

[43] Shen YC, Franz EA (2005). Hemispheric competition in left-handers on bimanual reaction time tasks. J. Mot. Behav..

[44] Taniguchi Y, Burle B, Vidal F, Bonnet M (2001). Deficit in motor cortical activity for simultaneous bimanual responses. Exp. Brain Res..

[45] Ekman M, Derrfuss J, Tittgemeyer M, Fiebach CJ (2012). Predicting errors from reconfiguration patterns in human brain networks. Proc. Natl. Acad. Sci. U. S. A..

