# Supplementary material for: Hand preference for the visual and auditory modalities in humans
Source: Sci Rep. 2021 Apr 12;11:7868. doi: 10.1038/s41598-021-87396-4 (PMC8041834; doi:10.1038/s41598-021-87396-4)
Supplement: Supplementary file 1 — Supplementary Information. [file 41598_2021_87396_MOESM1_ESM.pdf]

Supplementary Materials

Hand Preference for the Visual and Auditory Modalities in Humans

Yuqian Yang, Peter H. Weiss, Gereon R. Fink & Qi Chen*

Supplementary Results

S1. Robust visual dominance effects in terms of the PRP effect at the group level (n=119)

To test the potential interaction between hand dominance and the Psychological Refractory Period (PRP) effect in the visual vs. auditory modality, a 2 (sensory modality: visual vs. auditory) by 2 (response order: first vs. second response) by 2 (response hand: dominant vs. non-dominant hand) repeated measures ANOVA was performed. The main effect of the sensory modality was significant, $F_{(1,118)} = 68.44$, $p < 0.001$, $\eta_p^2 = 0.367$, indicating that RTs to the visual components of the bimodal trials (632 ± 10 ms) were significantly faster than RTs to the auditory components (650 ± 10 ms). The main effect of the response order was significant, $F_{(1,118)} = 245.64$, $p < 0.001$, $\eta_p^2 = 0.675$. The two-way interaction between the sensory modality and the response order was significant, $F_{(1,118)} = 26.07$, $p < 0.001$, $\eta_p^2 = 0.181$. Further planned *t*-tests on simple effects showed that it took longer time for the second auditory responses to recover from the PRP caused by the first visual responses (RTva_a > RTav_a = 133 ± 9 ms) than vice versa (RTav_v > RTva_v = 99 ± 7 ms), $t_{(118)} = 5.11$, $p < 0.001$, $d = 0.468$ (see Supplementary Fig. S1, right). However, the main effect of the response hand was not significant, $F_{(1,118)} = 1.81$, $p = 0.181$, $\eta_p^2 = 0.015$, suggesting that no significant RT difference was found between the dominant (640 ms) and non-dominant (642 ms) hands (see Supplementary Fig. S1, left). Neither the two-way interaction between the sensory modality and the response hand, $F_{(1,118)} = 0.74$, $p = 0.392$, $\eta_p^2 = 0.006$, nor the interaction between the response order and the response hand, $F_{(1,118)} = 0.47$, $p = 0.495$, $\eta_p^2 = 0.004$, was significant. The three-way interaction was also not significant, $F_{(1,118)} = 0.12$, $p = 0.727$, $\eta_p^2 = 0.001$. These results thus replicated the robust visual dominance effect in terms of the PRP effect. Also, neither a significant interaction between hand dominance and sensory dominance nor a significant dominant hand RT advantage was revealed at the whole group level.

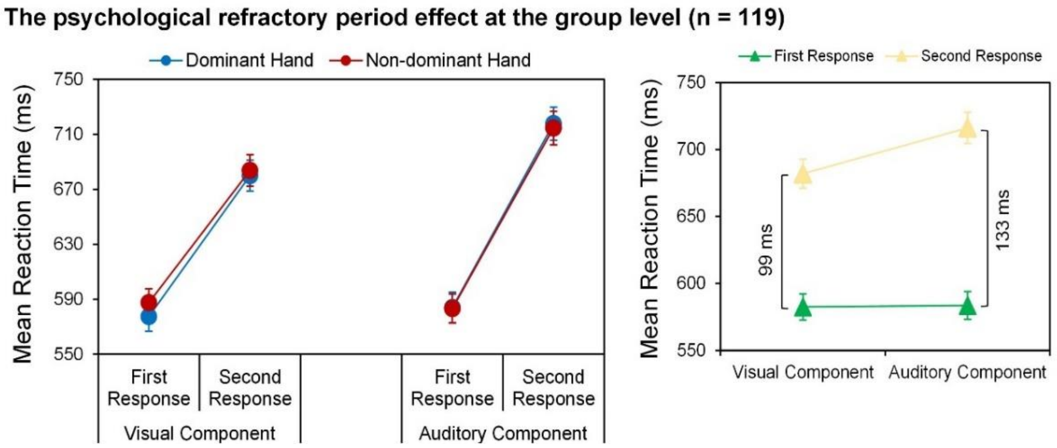

Supplementary Figure S1. Robust visual dominance effects in terms of the PRP effect at the group level (n = 119). Left panel, Mean RTs of the visual and the auditory components of the bimodal

trials are shown as a function of the sensory modality (visual vs. auditory), the response order (first vs. second response), and the response hand (dominant vs. non-dominant hand). **Right panel,** The simple effects of the PRP effect for the significant sensory modality*response order interaction. The error bars show standard errors (SEs).

S2. Interaction between the hand-modality preference and the PRP effect (sub-group data)

Visual Preference group (48% of participants, n=57)

RTs to the visual and auditory components of the bimodal trials were submitted to a 2 (hand-modality assignment: LARV vs. LVRA) by 2 (sensory modality: visual vs. auditory) by 2 (response order: first vs. second response) repeated-measures ANOVA to test how the hand-modality preference modulated the PRP effect. The main effect of the hand-modality assignment was significant, $F_{(1,56)} =$ 27.16, $p < 0.001$, $\eta_p^2 = 0.327$, indicating that bimodal RTs were significantly faster when the dominant hand was paired with the visual target (i.e., in the LARV condition, 637 ± 16 ms) than the auditory target (i.e., in the LVRA condition, 671 ± 16 ms). The main effect of sensory modality was significant, $F_{(1,56)} = 37.13$, $p < 0.001$, $\eta_p^2 = 0.399$, indicating that RTs to the visual components of the bimodal trials (646 ± 16 ms) were significantly faster than RTs to the auditory components (662 ± 16 ms). The main effect of the response order was significant, $F_{(1,56)} = 106.30$, $p < 0.001$, $\eta_p^2 = 0.655$. Moreover, the two-way interaction between the sensory modality and the response order was significant, $F_{(1,56)} = 10.28$, $p = 0.002$, $\eta_p^2 = 0.155$. Further planned *t*-tests on simple effects showed that, regardless of which sensory modality was assigned to the dominant hand, i.e., in both the LARV and LVRA conditions, it was significantly slower for the auditory responses to recover from the PRP induced by the previous visual responses (RTva_a > RTav_a = 123 ± 13 ms) than vice versa (RTav_v > RTva_v = 95 ± 10 ms), $t_{(56)} = 3.21$, $p = 0.002$, $d = 0.425$ (see Supplementary Fig. S2a, right). The two-way interaction between the hand-modality assignment and the response order also reached significance, $F_{(1,56)} = 5.83$, $p = 0.019$, $\eta_p^2 = 0.094$. Further planned *t*-tests on simple effects showed that the size of the PRP effect was significantly larger in the LARV condition (RTsecond > RTfirst = 118 ± 11 ms) than in the LVRA condition ((RTsecond > RTfirst = 100 ± 11 ms), $t_{(56)} = 2.42$, $p = 0.019$, $d = 0.320$ (see Supplementary Fig. S2a, right). These results suggested that, when the dominant hand was used for visual responses (i.e., in the LARV condition), if one modality lost the multisensory competition for the visual preference group, it became more difficult to overcome the PRP caused by the other modality. Neither the two-way interaction between the hand-modality assignment and the sensory modality, $F_{(1,56)} = 2.50$, $p = 0.120$, $\eta_p^2 = 0.094$, nor the three-way interaction, $F_{(1,56)} = 0.19$, $p = 0.665$, $\eta_p^2 = 0.003$, was significant.

Auditory Preference group (39% of participants, n=47)

RTs to the visual and auditory components of the bimodal trials were submitted to a 2 (hand-modality assignment: LARV vs. LVRA) by 2 (sensory modality: visual vs. auditory) by 2 (response order: first vs. second responses) repeated-measures ANOVA to test how the hand-modality preference modulated the PRP effect. The main effect of the hand-modality assignment was significant, $F_{(1,46)} = 18.96$, $p < 0.001$, $\eta_p^2 = 0.292$, indicating that responses in the bimodal condition were significantly faster when the dominant hand was assigned to the auditory target (i.e., in the LVRA condition, 610 ± 13 ms) than when the dominant hand was assigned to the visual target (i.e., in the LARV condition, 640 ± 15 ms). The main effect of the sensory modality was significant, $F_{(1,46)} = 25.58$, $p < 0.001$, $\eta_p^2 = 0.357$, indicating that RTs to the visual components of the bimodal trials (617 ± 13 ms) were significantly faster than RTs to the auditory components (633 ± 14 ms). The main effect of the response order was also significant, $F_{(1,46)} = 99.73$, $p < 0.001$, $\eta_p^2 = 0.684$. Moreover, the two-way interaction between the sensory modality and the response order was significant, $F_{(1,46)} = 10.76$, $p = 0.002$, $\eta_p^2 = 0.190$. Further planned *t*-tests on simple effects suggested that regardless of which sensory modality was assigned to the dominant hand, i.e., in both the LARV and LVRA conditions, it was significantly slower for the auditory responses to recover from the PRP induced by the previous visual responses (RTva_a > RTav_a = 142 ± 16 ms) than vice versa (RTav_v > RTva_v = 105 ± 10 ms), $t_{(46)} = 3.28$, $p = 0.002$, $d = 0.479$ (see Supplementary Fig. S2b, right). The two-way interaction between the hand-modality assignment and the response order also reached significance, $F_{(1,46)} = 8.56$, $p = 0.005$, $\eta_p^2 = 0.157$. Further planned *t*-tests on simple effects showed that the size of the PRP effect was significantly larger in the LVRA condition (RTsecond > RTfirst = 135 ± 14 ms) than in the LARV condition (RTsecond > RTfirst = 112 ± 12 ms), $t_{(46)} = 2.93$, $p = 0.005$, $d = 0.427$ (see Supplementary Fig. S2b, right). Therefore, during the multisensory competition, it took more time for the losing modality to overcome the PRP effect caused by the winning modality when the dominant hand was assigned to the auditory target (i.e., in the LVRA condition) for the auditory preference group. Neither the two-way interaction between the hand-modality assignment and the sensory modality, $F_{(1,46)} = 0.01$, $p = 0.924$, $\eta_p^2 = 0.000$, nor the three-way interaction, $F_{(1,46)} = 0.01$, $p = 0.934$, $\eta_p^2 = 0.000$, was significant.

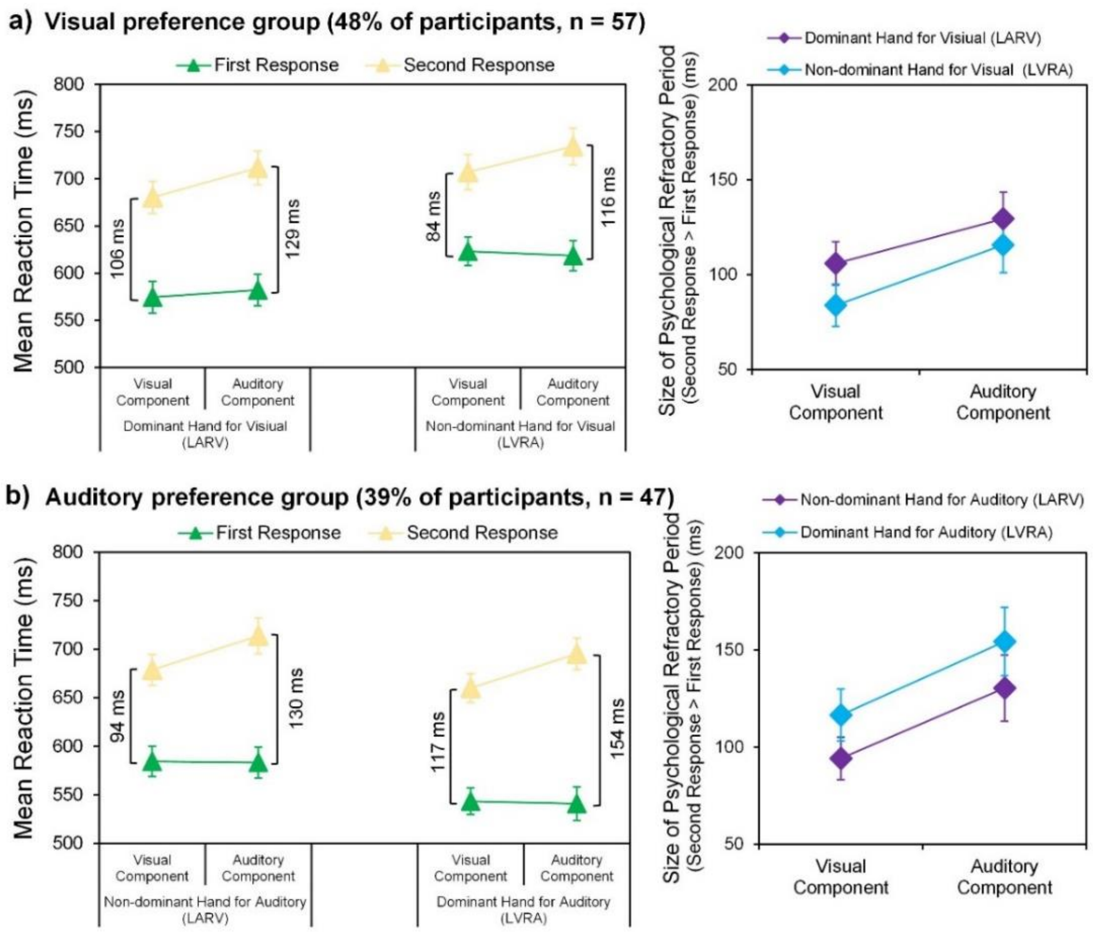

Supplementary Figure S2. Interaction between the hand-modality preference and the PRP effect for the *visual* preference group (a) and the *auditory* preference group (b). Left panel, Bimodal RTs are shown as a function of the sensory modality (visual vs. auditory) and the response order (first vs. second response) for the LARV (left part) and LVRA (right part) conditions. **Right panel,** The simple effects for the significant sensory modality*response order interaction. The sizes of the PRP effect for the visual components (RTav_v > RTva_v) and the auditory components (RTva_a > RTav_a) are shown as a function of hand-modality assignment (LARV vs. LVRA). The error bars show standard errors (SEs).

Taken together, both groups showed a significant visual dominance effect in terms of the PRP effect. Regardless of the type of hand-modality assignment, it took longer for audition to recover from the PRP effect caused by vision during the multisensory competition (see Supplementary Figs. S2a and S2b, right). Furthermore, the size of the PRP effect could be modulated by the individual hand-modality preference. When the hand-modality assignment was congruent with the specific hand-modality preference of each group (i.e., in the LARV condition for the visual preference group and the LVRA condition for the auditory preference group), the PRP effect was boosted, making it more difficult for the losing modality to overcome the PRP caused by the winning modality (see Supplementary Figs. S2a and S2b, right).

S3. Interaction between the hand-modality preference and the proportions of the VA vs. AV bimodal trials (sub-group data)

Visual Preference group (48% of participants, n=57)

Proportions of the VA and AV trials were submitted to a 2 (hand-modality assignment: LARV vs. LVRA) by 2 (sensory dominance: VA vs. AV trials) repeated-measures ANOVA to test how the hand-modality preference modulated the sensory dominance effect in terms of proportion data. The main effect of the sensory dominance was the only significant effect ($F_{(1,56)} = 8.28$, $p = 0.006$, $\eta_p^2 = 0.129$): the proportion of the VA trials (44% ± 1%) was significantly higher than the proportion of the AV trials (38% ± 1%, see Supplementary Fig. S3a). Neither the main effect of the hand-modality assignment ($F_{(1,56)} = 0.01$, $p = 0.907$, $\eta_p^2 = 0.000$), nor the interaction ($F_{(1,56)} = 1.72$, $p = 0.196$, $\eta_p^2 = 0.030$) were significant. Therefore, regardless of which sensory modality was paired with the dominant hand, vision won the multisensory competition more frequently than audition, indicating a significant visual dominance effect for the visual preference group.

Auditory Preference group (39% of participants, n=47)

Proportions of the VA and AV trials were submitted to a 2 (hand-modality assignment: LARV vs. LVRA) by 2 (sensory dominance: VA vs. AV) repeated-measures ANOVA to test how the hand-modality preference modulated the sensory dominance effect in terms of proportion data. The main effect of the sensory dominance was significant ($F_{(1,46)} = 14.58$, $p < 0.001$, $\eta_p^2 = 0.241$): the proportion of the VA trials (48% ± 2%) was significantly higher than the proportion of the AV trials (36% ± 2%, see Supplementary Fig. S3b). Neither the main effect of the hand-modality assignment ($F_{(1,46)} = 0.42$, $p = 0.519$, $\eta_p^2 = 0.009$), nor the interaction ($F_{(1,46)} = 0.21$, $p = 0.649$, $\eta_p^2 = 0.005$) were significant. Therefore, irrespective of which sensory modality was assigned to the dominant hand, visual responses dominated auditory responses more frequently, indicating the dominance of vision over audition for the auditory preference group.

Taken together, despite their different hand-modality preferences, both groups showed a robust visual dominance effect indicated by a significantly higher frequency of those bimodal trials, in which the visual responses preceded the auditory responses (VA trials) than vice versa (AV trials; see Supplementary Figs. S3a and S3b).

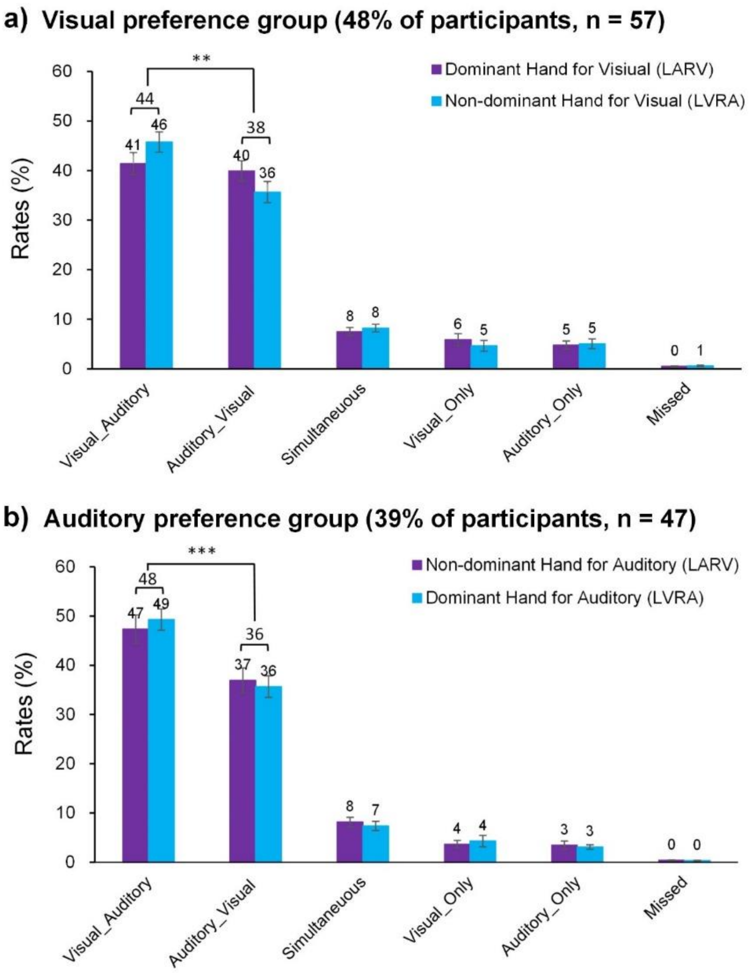

Supplementary Figure S3. Proportions of the six different types of conditions in the bimodal trials are shown as a function of the hand-modality assignment (LARV vs. LVRA) in the *visual* preference group (a) and the *auditory* preference group (b). ***$p < 0.001$, **$p < 0.01$, *$p < 0.05$. The error bars show standard errors (SEs).
